# Circulating microRNAs as minimal residual disease biomarkers in childhood acute lymphoblastic leukemia

**DOI:** 10.1186/s12967-019-2114-x

**Published:** 2019-11-14

**Authors:** Andrea Rzepiel, Nóra Kutszegi, András Gézsi, Judit C. Sági, Bálint Egyed, György Péter, Henriett Butz, Gábor Nyírő, Judit Müller, Gábor T. Kovács, Csaba Szalai, Ágnes F. Semsei, Dániel J. Erdélyi

**Affiliations:** 1 2nd Department of Paediatrics, Semmelweis University, Budapest, Hungary; 2 Department of Genetics, Cell- and Immunobiology, Semmelweis University, Budapest, Hungary; 3 MTA-SE Immune-Proteogenomics Extracellular Vesicle Research Group, Budapest, Hungary; 4 Department of Measurement and Information Systems, Budapest University of Technology and Economics, Budapest, Hungary; 5 Heim Pál Children’s Hospital, Budapest, Hungary; 6 Department of Laboratory Medicine, Semmelweis University, Budapest, Hungary; 7 MTA-SE Molecular Medicine Research Group, Hungarian Academy of Sciences and Semmelweis University, Budapest, Hungary

**Keywords:** MicroRNA, Biomarker, Pediatric acute lymphoblastic leukemia, Minimal residual disease

## Abstract

**Background:**

Treatment stratification based on bone marrow minimal residual disease (MRD) at set time points has resulted in considerably improved survival in pediatric acute lymphoblastic leukemia (ALL). Treatment response is assessed using bone marrow samples. MicroRNAs (miRs) easily traffic among fluid spaces and are more stable than most other RNA classes. We examined the role of circulating miRs as putative less invasive MRD biomarkers.

**Methods:**

In an exploratory experiment, expression of 46 preselected miRs was studied in platelet-free blood plasma samples of 15 de novo, 5 relapsed ALL patients and 10 controls by Custom TaqMan Array Advanced MicroRNA Card. Based on their high expression in ALL compared to controls, and on the reduction observed along the induction therapy, four miRs were selected for further analyses: miR-128-3p, -181a-5p, -181b-5p and 222-3p. Their expression was measured by qPCR at 4 time points in 27 de novo ALL patients treated in the ALL IC-BFM 2009 study.

**Results:**

The expression of all 4 miRs significantly decreased over the first week of therapy (miR-128-3p: log_2_ fold change − 2.86; adjusted p 3.6 × 10^−7^; miR-181b-5p: log_2_ fold change − 1.75; adjusted p 1.48 × 10^−2^; miR-181a-5p: log_2_ fold change -1.33; adjusted p 3.12 × 10^−2^; miR-222-3p: log_2_ fold change − 1.25; adjusted p 1.66 × 10^−2^). However, no significant further reduction in miR expression was found after the 8th day of therapy. Measured drop in expression of 2 miRs at day 8 strongly correlated with day 15 bone marrow flow cytometry MRD results (miR-128-3p: Pearson’s r = 0.88, adjusted p = 2.71 × 10^−4^; miR-222-3p: r = 0.81, adjusted p = 2.99 × 10^−3^).

**Conclusion:**

In conclusion, these circulating miRs might act as biomarkers of residual leukemia. MiR-128-3p and miR-222-3p in blood predict day 15 flow cytometry MRD results 7 days earlier. Although, their sensitivity falls behind that of bone marrow flow cytometry MRD at day 15.

## Background

Treatment stratification based on early response to therapy greatly improved survival in pediatric acute lymphoblastic leukemia (ALL). Treatment response is assessed as quantified minimal residual disease (MRD) in bone marrow (BM) at set time points. For the last one or two decades, MRD assessment has been part of the routine clinical practice in the developed world. Flow cytometry and/or qPCR based methods are used in various countries, with sensitivities of 10^−3^–10^−4^ and 10^−4^–10^−6^, respectively [1]. Currently, application of high throughput sequencing methods are being studied to increase MRD sensitivity and to improve patient stratification [2]. Compared to individual IgH and TCR qPCR testing, next generation sequencing would also save working hours of laboratory staff. Whichever method is used, children are put under general anesthesia (GA) and undergo BM aspiration for MRD follow up 2–5 times following their diagnosis. This is because if little residual leukemia is left, it is most reliably and abundantly found in the BM [1]. GA and marrow aspiration, together with preparations and related issues (e.g. consenting, starving for anesthesia, checks, theatre lists, observations post procedure) put quite a burden on children, their families and on hospital staff.

MicroRNAs (miRs) are small non-coding RNAs, ranging from 19 to 25 nucleotides in size [3]. Their role is in posttranscriptional regulation of mRNA expression. MiRs regulate about half of the human genes [4]. Binding of miRs to their target sequence, with perfect complementarity in seed region and base paring in central region, results in mRNA cleavage and degradation. In case of less complementarity, translation repression is their typical function [5]. There are over 2000 miRs known in human [6]. The miR profile is distinct in every body fluid. In the blood, for example, platelets have a repertoire of approximately 750 miRs [7, 8], with the let-7 family contributing in almost 50% [8]. MiRs can be tissue specific regarding their origin, or there are examples when certain miRs are expressed at specific stages of ontogenesis or specific stages of cell differentiation [9, 10].

MiR profiles in normal tissues and in different diseases are intensively studied despite of numerous pitfalls [11]. MiRs are actively secreted into the extracellular space via extracellular vesicles [12]. The secreted miRs partly reflect the intracellular miR signature of the source cells, but certain miRs are withheld or selectively excreted in higher amounts [13]. Compared to mRNAs, miRs are much more stable. Some of them persist for 5 days [14, 15]. MiRs were found to be protected from RNases by encapsulation into extracellular vesicles, by complexes formed with Argonaute proteins or Nucleophosmin-1 and by their uptake into lipoproteins [16–18]. As a very universal phenomenon, malignant cells secrete higher amounts of miRs than healthy cells or tissues [19]. This, together with the selective over-secretion of some miRs, such as miR-128-3p [20–22], their easy distribution among fluid spaces and their stability makes miRs good candidates for cancer biomarkers. Indeed, very promising such results were published in solid tumors, e.g. in breast cancer and non-small cell lung cancer [23]. Some similar biomarker studies were published in leukemia, too. These focused on the miR signatures in BM, peripheral blood (PB) or plasma taken at the time of diagnosis as biomarkers of leukemia diagnosis, leukemia subtype, with disease characteristics or predictors of prognosis [24–26].

Our aim was to evaluate circulating miRs as biomarkers for MRD monitoring in pediatric ALL. Compared to the current practice of MRD monitoring, this approach would have the great advantage of less invasive sampling and no need for GA. Our strategy was to (1) get serial patient plasma samples along the induction cycle prospectively, (2) discard platelets very early as the largest source of miRs in the blood, (3) choose miRs for analyses based on the previously described miR profile of leukemic BM (4) for our first analysis on an exploratory cohort with array, and (5) proceed to validation of the selected best candidate biomarkers with qPCR on an extended patient cohort.

## Methods

### Patients and samples

We studied 28 pediatric patients with de novo and 5 patients with 1st relapse of precursor-B cell ALL, age 1–18 years, diagnosed between 2016 and 2018 at the 2nd Department of Pediatrics, Semmelweis University or at Heim Pál Children’s Hospital, both in Budapest, Hungary. Informed consent was requested from legal guardians of the patients. The study was approved by the Ethics Committee of the Hungarian Medical Research Council (60106-1/2015/EKU and 6886/2019/EKU) and conducted according to the principles of the Declaration of Helsinki. De novo ALL patients from only three cytogenetic subgroups were included: those with hyperdiploidy (blast chromosome number ≥ 47), those with t(12;21) (p13;q22) alias ETV6/RUNX1 fusion and those with no known cytogenetic abnormality (normal findings on a set FISH panel targeting frequent ALL rearrangements, found to be diploid by flow cytometry DNA index, either normal karyotype or unsuccessful karyotyping). Karyotype of relapsed patients was not used as selection criteria or for grouping because of the limited number of available samples.

Peripheral blood (PB) (3.5 ml) of patients was collected at diagnosis and also at days 8, 15 and 33 of the induction cycle of the ALL IC-BFM 2009 trial protocol. Bone marrow (BM) aspirates (1 ml) were collected at diagnosis before any treatment and on days 15 and 33. Blood and bone marrow samples of relapsed patients were collected only at diagnosis. In addition, control PB samples were collected from non-leukemic subjects. Control patients had one of the following conditions: vitamin D deficiency, otitis media, impetigo, hypothyreosis, neurofibromatosis type 1, iron deficiency anemia, phimosis.

Preparation of platelet-free plasma (PFP) was carried out within 2 h of sampling and was based on the recommendation of the International Society of Thrombosis and Haemostasis [27]. Sodium citrate tubes were used for all sample types. Samples taken at diagnosis were collected before any steroid or cytostatic treatment would have been started for the patient. The use of PFP was chosen based on the fact that platelets have a large repertoire of miRs and are the major source of miRs in plasma [28]. Whole blood and bone marrow samples were centrifuged at 2500×*g* for 15 min at 16 °C. Supernatant was transferred into a new centrifuge tube and centrifuged again for 15 min at 16 °C 2500×*g*. Supernatant was aliquoted immediately and stored at − 80 °C until use.

Clinical data were collected during the first month of therapy: white blood count at diagnosis, absolute blast number at day 8 in peripheral blood, bone marrow flow cytometry (FC) MRD at day 15, absolute white blood cell count at day 33, absolute lymphocyte count at day 33 and FC MRD at day 33. FC MRD was measured in routine diagnostics as per the standards of ALL IC-BFM 2009 study.

Detailed description of patients and samples used is provided in Table 1, 2, and 3.

**Table 1:** Samples analyzed on TaqMan low density array and with advanced qPCR

Subgroups	Discovery population				Extended population			
	BM at Day 0 (n)	PB at Day 0 (n)	PB at Day 8 (n)	PB at Day 33 (n)	PB at Day 0 (n)	PB at Day 8 (n)	PB at Day 15 (n)	PB at Day 33 (n)
De novo ALL								
Hyperdiploid	3	3	3	2	8	8	8	8
t(12;21)	6	6	3	2	9	9	9	9
Normal^a^	6	6	3	2	10	10	10	9
Relapsed ALL	5	5						
Control		10			10			
SUM	20	30	9	6	37	27	27	26

Sampling dates are marked as days of the induction chemotherapy cycle

*BM* bone marrow, *PB* peripheral blood

^a^Normal karyotype: no alteration on FISH, DNA index diploid and cytogenetics normal or unsuccessful

**Table 2:** Characteristics of the population studied on custom TaqMan low density array

Subgroups	Age in years median (range)	Number of patients (n)	Male (n)	Female (n)
De novo ALL				
Karyotype: hyperdiploid	3.1 (2.0–3.7)	3	1	2
t(12;21)	3.6 (3.1–4.6)	6	3	3
Karyotype: normal^a^	3.1 (1.5–5.3)	6	4	2
Relapsed ALL	10.4 (6.3–18.8)	5	3	2
Total number of patients	3.9 (1.5–18.8)	20	11	9
Control	4.4 (1.6–15.1)	10	6	4

^a^Normal: no alteration on FISH, DNA index diploid and cytogenetics normal or unsuccessful

**Table 3:** Characteristics of the population studied by advanced qPCR method

Subgroups	Age in years median (range)	Number of patients (n)	Male (n)	Female (n)
De novo ALL				
Karyotype: hyperdiploid	3.8 (2.0–13.7)	8	1	7
t(12;21)	3.1 (2.2–4.6)	9	6	3
Karyotype: normal^a^	4.9 (1.5–17.3)	11	9	2
Control	4.4 (1.6–15.1)	10	6	4

^a^Normal: no alteration on FISH, DNA index diploid and cytogenetics normal or unsuccessful

### RNA isolation, cDNA synthesis and miR profiling on array

With the aim to find MRD biomarkers, 46 miRs were selected for analysis. Only miRs found to be overexpressed in blasts in at least two different previously published studies were chosen (Table 4).

**Table 4:** The 46 microRNAs selected for custom TaqMan low density array

MicroRNA	Field of research	References
Cel-miR-39	Exogenous, spike-in control	[29, 30]
miR-103a-3p, miR-638 miR-484, miR-30d-5p, miR-16-5p	Candidate reference microRNAs	[31–33]
let-7a-5p, let-7f-5p	Platelet origin microRNAs	[8]
miR-128-3p, miR-181b-5p	Bone marrow cells	[34, 35]
miR-222-3p, miR-223-3p, miR-361-3p, miR-374a-5p, miR-501-5p, miR-511-5p, miR-532-5p, miR-660-5p, miR-98-5p	Hyperdiploid ALL	[36]
miR-20a-5p, miR-93-5p	Different ALL cell lines	[37]
miR-100-5p, miR-125b-5p, miR-126-3p, miR-320a, miR-383-5p, miR-494-3p, miR-629-5p, miR-99a-5p	t(12;21) ALL	[31, 35, 36, 38]
miR-17-5p, miR-181a-5p, miR-181b-5p, miR-181c-5p, miR-320a, miR-342-3p, miR-106a-5p, miR-1290	Relapsed ALL	[34, 35, 38–40]
miR-146a-5p, miR-155-5p, miR-222-3p, miR-34a-5p, miR-511-5p, miR-99a-5p	Circulating microRNAs in pre-B ALL	[31]
miR-632, miR-654-5p	T and dendritic cell lines	[18]

MiRNeasy Serum/Plasma Kit (Qiagen, Hilden, Germany) was used for RNA isolation from defrosted PFP samples. Synthesis of cDNA was carried out using TaqMan Advanced miRNA cDNA Synthesis Kit (Thermo Fisher Scientific, Waltham, MA, USA) according to the protocol supplied by the manufacturer.

MiR profiling of 65 PFP samples from 20 ALL patients (15 de novo and 5 relapsed ALL) and 10 control samples was performed using Custom TaqMan Advanced Low Density miRNA Array (TLDA) Card (Thermo Fisher Scientific, Waltham, MA, USA). Measurements were carried out using QuantStudio 7 Flex Real-Time PCR System (Thermo Fisher Scientific, Waltham, MA, USA). GeNorm algorithm was used to calculate the gene expression stability measure for each potential reference miR, based on the average pairwise variation between all candidate reference miRs. As a validation, NormFinder algorithm was also used which takes intra- and inter-group variability into account. Based on these analyses, miR-484 was selected for normalizing, and the ΔCt value was calculated for each miR. Four samples measured in duplicates on TLDA Card and correlated very highly (Pearson’s r = 0.981 (CI 95% 0.974–0.986), p < 10^−16^).

### Quantitative real-time PCR

Based on the results of TLDA cards, 4 candidate miRs were selected for further investigation on an extended population using Advanced qPCR methodology. Quantitative Real-time PCR TaqMan Advanced miRNA assays were used according to the manufacturer’s instruction (Thermo Fisher Scientific, Waltham, MA, USA). Thermo Fisher 7900HT Fast Real-Time PCR system was used for real-time quantitative PCR measurement (Thermo Fisher Scientific, Waltham, MA, USA). PFP samples originated from peripheral blood of 28 patients with ALL and 10 controls were measured, in total 110 samples (Table 3 for details). Detection of 5 miRs (miR-181a-5p, miR-81b-5p, miR-128-3p, miR-22-3p and control miR-484) were performed in duplicates (correlation between duplicates was calculated: Pearson’s r = 0.998, p < 2.2*10^−16^). Correlation between Advanced qPCR and TLDA measurements was: Pearson’s r = 0.873, p = 3.19*10^−44^.

### Statistical analysis

All statistical analyses were performed using R statistical software (R Foundation for Statistical Computing, Vienna, Austria; version 3.5.1). To select suitable reference miRs for normalization, the geNorm and NormFinder algorithms of the NormqPCR [41] package was used. Statistical differential expression of miRs was determined by the Limma [42] package. For that, a linear model was fitted for each gene based on the subgroup of the patients or the different sampling times. The gender of the patients was used as a coefficient in both cases. Then, moderated t-statistics and log-odds of differential expression were calculated by empirical Bayes moderation of the standard errors towards a common value. The resulting p-values obtained were corrected for multiple testing using the Benjamini–Hochberg procedure. Genes were considered to be differentially expressed when the adjusted p-value false discovery rate was below 0.05. Pearson correlation coefficients were calculated to evaluate the correlation between the flow cytometry MRD results and the log_2_ transformed expression level or fold change of miRs, respectively. In order to deal with multiple comparisons, the p-values were adjusted by the Benjamini–Hochberg false discovery rate method with type I error rate of 5%.

## Results

### MiR expression pattern of the exploratory cohort on array

**Fig. 1:**
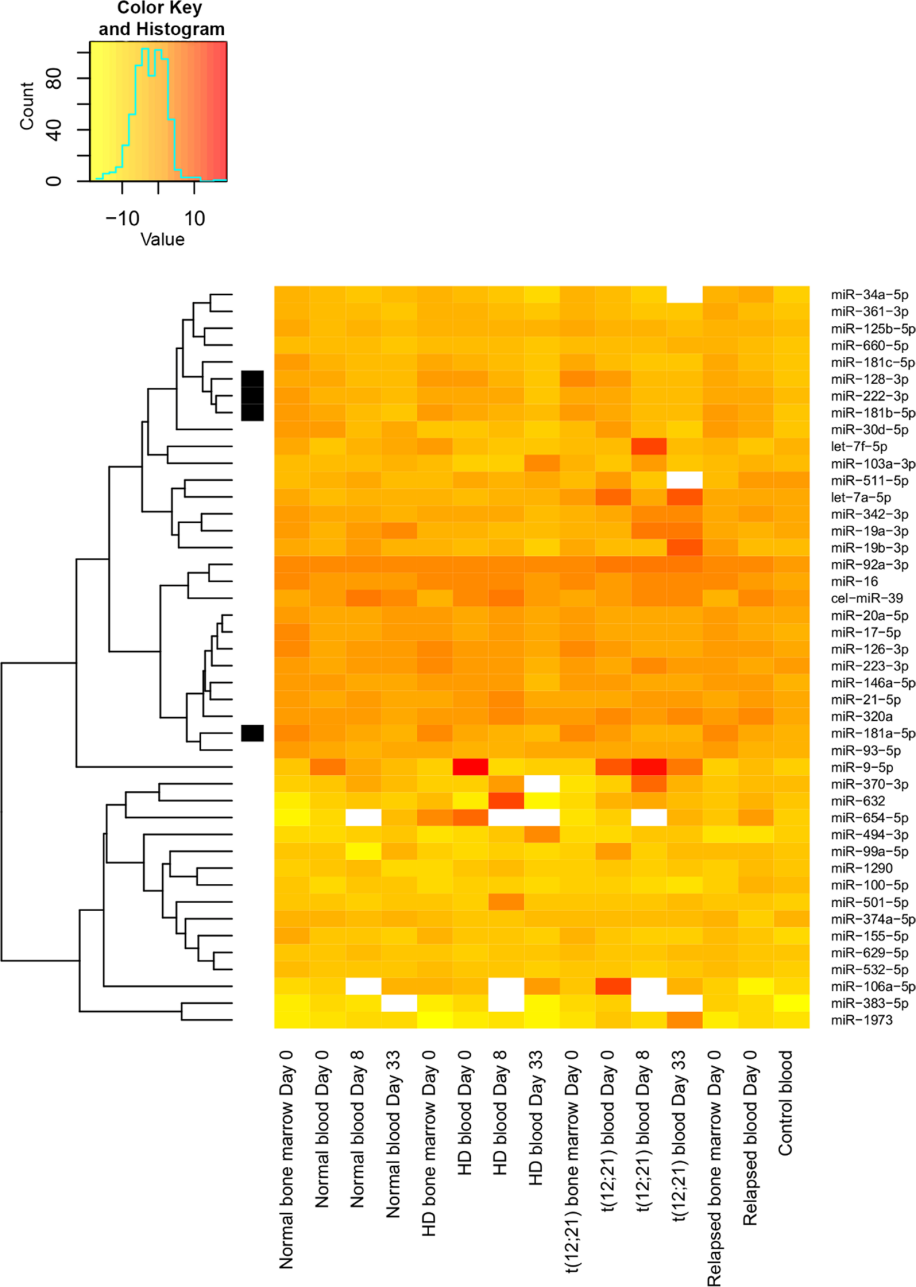
TaqMan low density array miR expression, comparison of ALL subgroups and controls. The heatmap displays the expression pattern of microRNAs measured in platelet-free plasma derived from bone marrow and peripheral blood samples of pediatric ALL patients. The horizontal axis shows the origin of samples in different subgroups (normal karyotype: no alteration on FISH, DNA index diploid and cytogenetics normal or unsuccessful; HD: hyperdiploid subgroup), the day of sampling in relation to start of treatment. The vertical axis shows the 44 previously selected microRNA repertoire. miR-484 was used as normalizing miR. Expression of further 2 miRs was not detected in any samples. The scale shows the normalized log_2_ transformed expression levels. Yellow indicates microRNA down-expression, red indicates overexpression compared to the normalizing miR

**Fig. 2:**
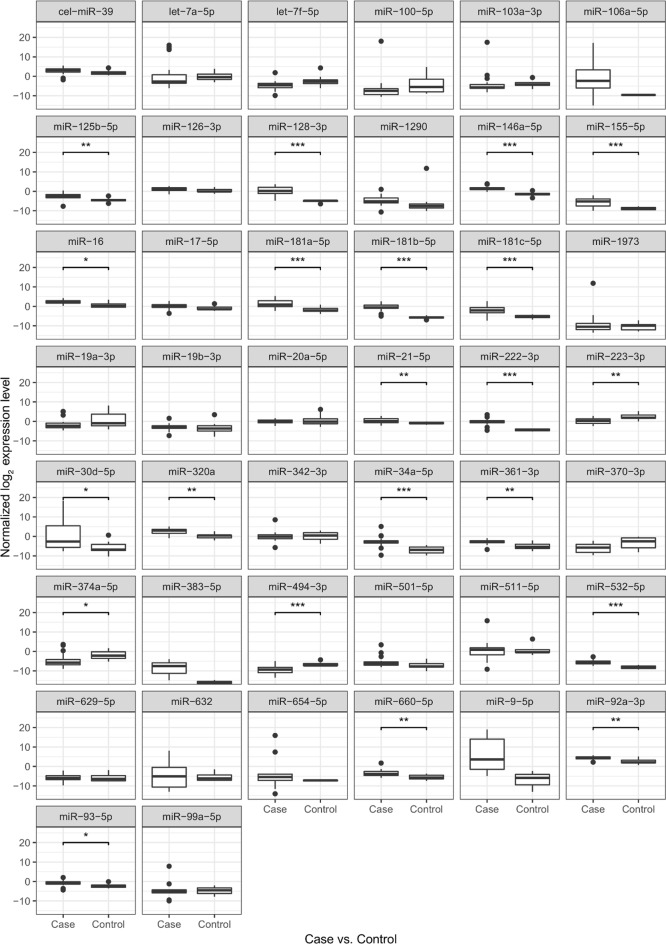
MiR expression pattern in ALL and control PB PFP samples measured by TLDA. Case indicates de novo and relapsed ALL day 0 PB PFP samples, control indicates non-leukemic PB PFP samples. Data obtained on TLDA measurements. Box: the 2nd and 3rd quartiles; thick line in the box: median; lower whiskers: minimal value if there are no low outrange values, or Q1 − 1.5*IQR; upper whiskers: maximum value if there is no upper outlier, or Q3 + 1.5*IQR; dots: outliners, lines above the boxes: significant correlation (p < 0.05); ***p value: 0–0.001; **p value: 0.001–0.01; *p value: 0.01–0.05

**Fig. 3:**
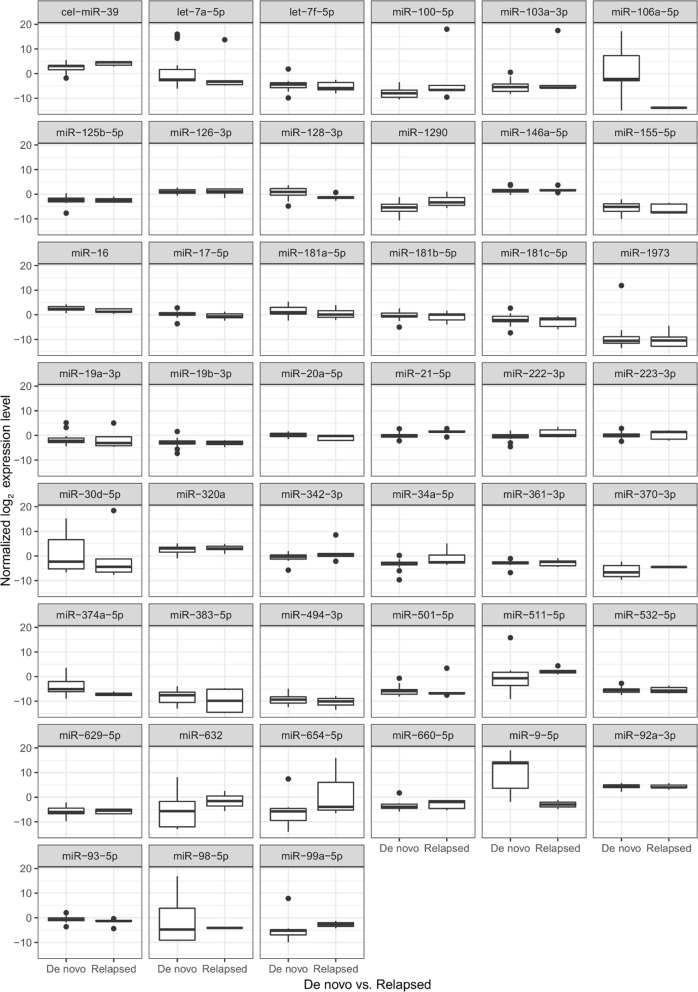
MiR expression in de novo and relapsed ALL PB PFP at the day of diagnosis (by TLDA). Box: the 2nd and 3rd quartiles; thick line in the box: median; lower whiskers: minimal value if there are no low outrange values, or Q1 − 1.5*IQR; upper whiskers: maximum value if there is no upper outlier, or Q3 + 1.5. No significant differences were found

Expressions of 46 selected miRs were measured in PFP samples of de novo and relapsed pediatric precursor-B acute lymphoblastic leukemia patients and controls on custom TaqMan low density array (TLDA). See the results illustrated in Figs. 1, 2, and 3. There was no significant correlation between miR expressions of the bone marrow and the peripheral blood (adjusted p > 0.05, data not shown).

The expressions of 19 miRs in ALL PB PFP at diagnosis were significantly different compared to the control samples (p < 0.05). In line with our selection criteria, 18 miRs showed higher expression, while one miR showed downregulation in leukemic patients (Fig. 2). Different karyotype (hyperdiploid, t(12, 21), normal) subgroups were separately compared to the control PFP samples. Five miRs were significantly upregulated in every subgroup: miR-128-3p, miR-146a-5p, miR-181b-5p, miR-222-3p and miR-532-5p (p < 0.05). There was no unique miR expression pattern in subgroups of patients based on our TLDA measurements. Platelet originated miRs (let-7a-5p and let-7f-5p) were not significantly upregulated or downregulated compared to the control samples.

When miR expression differences were analysed between day 0 and day 33 of therapy in PB PFP, 4 miRs showed significantly lower expression at day 33: miR-128-3p, miR-181a-5p, miR-181b-5p, miR-222-3p. These 4 miRs were further investigated by Advanced qPCR on the extended cohort.

### MiR expression pattern of the extended cohort by advanced qPCR

Relative expression levels of the 4 selected miRs were first compared between ALL day 0 PB PFP samples and PB PFP of non-leukemic controls. ALL subgroups of different karyotypes were examined separately as well as the whole ALL group (Table 5).

**Table 5:** miR expressions in diagnostic ALL samples compared to controls (PB PFP samples, advanced qPCR results)

Subgroups	miR-128-3p log_2_FC (adj. p value)	miR-181a-5p log_2_FC (adj. p value)	miR-181b-5p log_2_FC (adj. p value)	miR-222-3p log_2_FC (adj. p value)
Karyotype: hyperdiploid	3.39 (7.05*10^−4^)	NS	NS	2.09 (4.06*10^−2^)
Karyotype: t(12;21)	5.93 (9.59*10^−10^)	2.56 (1.38*10^−2^)	3.84 (2.26*10^−4^)	3.34 (2.21*10^−4^)
Karyotype: normal^a^	4.23 (1.78*10^−5^)	3.45 (5.35*10^−4^)	4.31 (4.73*10^−5^)	2.63 (4.76*10^−3^)
Whole cohort	4.52 (3.48*10^−9^)	2.46 (2.11*10^−3^)	3.46 (4.73*10^−5^)	2.68 (2.15*10^−4^)

Values in the table indicate the average log_2_ fold change between cases and controls

*NS* no significant correlation

^a^Normal karyotype: no alteration on FISH, DNA index diploid and cytogenetics normal or unsuccessful

Changes of PB PFP miR expression along the induction cycle were analyzed in paired tests. Higher expression of all 4 miRs identified on TLDA (miR-128-3p, miR-181a-5p, miR-181b-5p and miR-222-3p), was validated in the extended study population.

**Fig. 4:**
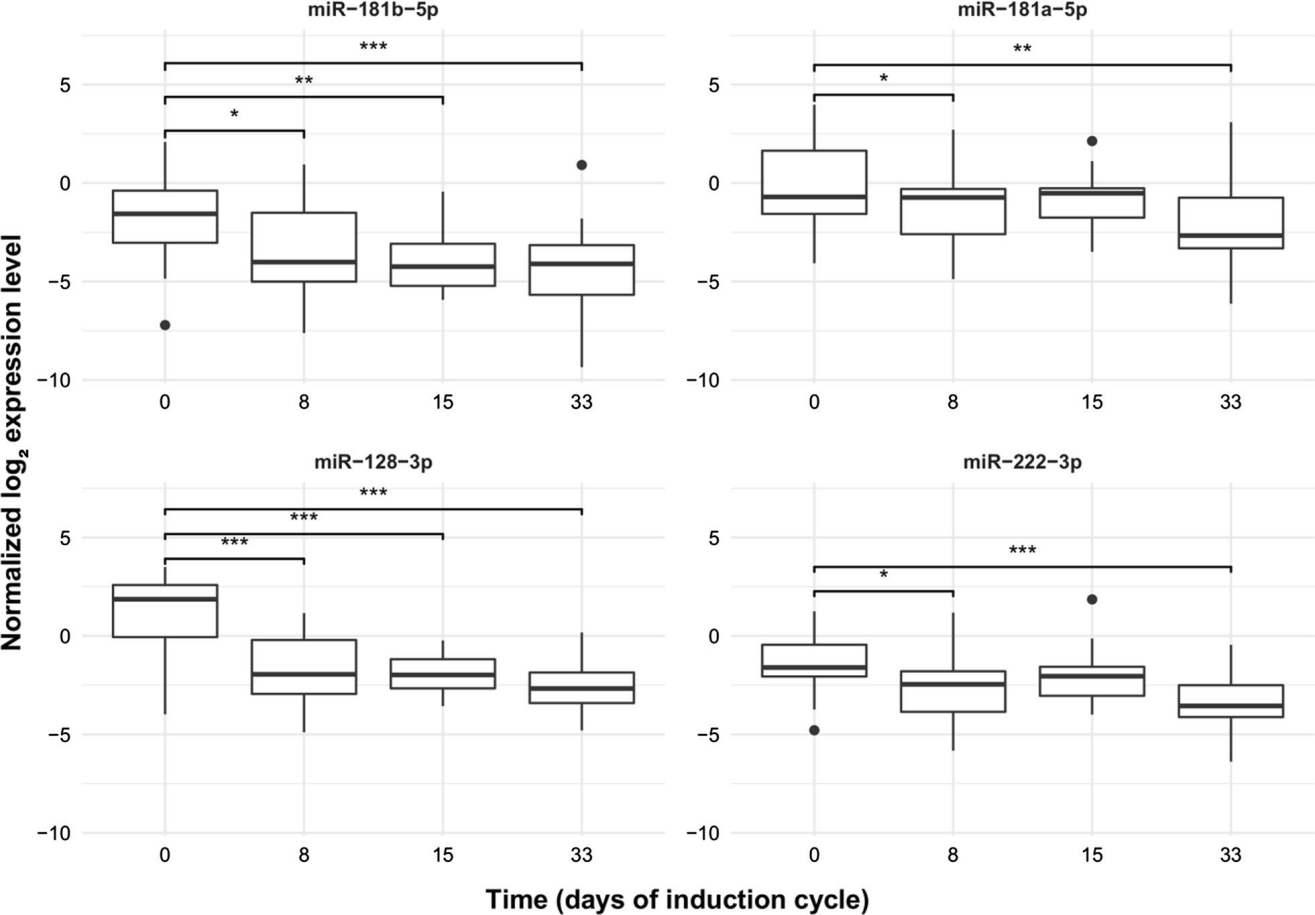
MiR expressions in PB PFP during the first month of therapy (by TaqMan Advanced qPCR). Box: the 2nd and 3rd quartiles; thick line in the box: median; whiskers: minimum and maximum values if there are no outrange values, or Q1 − 1.5*IQR; dots: outliers, lines above the boxes: significant correlation (p < 0.05); ***p value: 0 ≤ p < 0.001, **p value: 0.001 ≤ p < 0.01, *p value: 0.01 ≤ p < 0.05

**Fig. 5:**
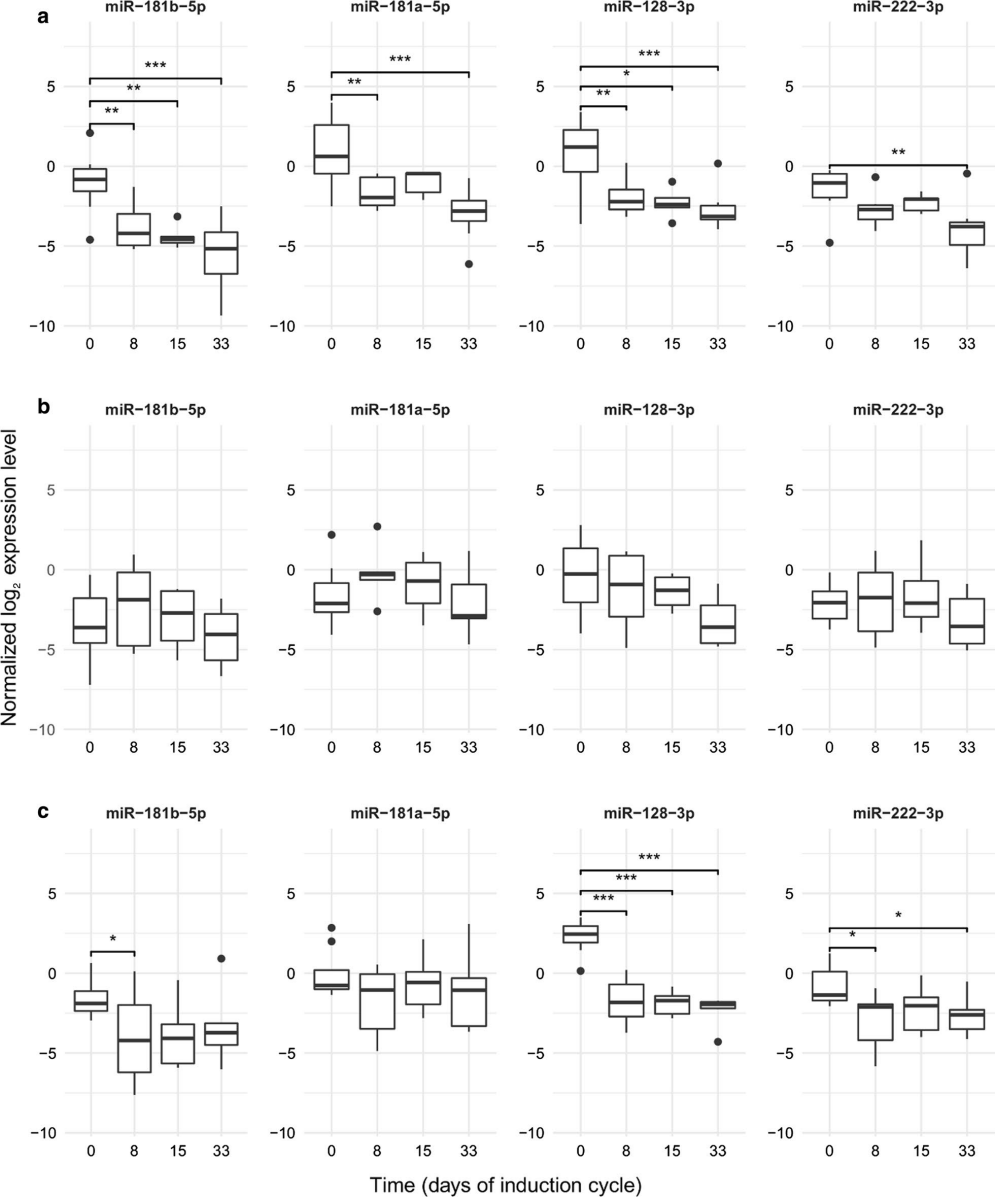
MiR expression during the first month of the therapy in cytogenetic subgroups of ALL (by qPCR). **a** Normal karyotype (no alteration on FISH, DNA index diploid and cytogenetics normal or unsuccessful); **b** hyperdiploid subgroup; **c** t(12;21). Box: the 2nd and 3rd quartiles; thick line: median; lower whiskers: minimal value if there are no low outlier values, or Q1 − 1.5*IQR; upper whiskers: maximum value if there is no upper outlier, or Q3 + 1.5*IQR; dots: outliners. Lines above the boxes: significant correlation (p < 0.05); ***p value: 0–0.001, **p value: 0.001–0.01, *p value: 0.01–0.05

Measured by qPCR in this larger cohort, the expression of all four miRs decreased significantly after the start of treatment. However, no further significant drop could be demonstrated after day 8 with regards to any of the miRs (Fig. 4; Table 6). This question was also explored in the three ALL cytogenetic subgroups separately, and similar trends were found (Fig. 5). The best correlations and tendencies were seen with miR-181b-5p, miR-128-3p expression within the normal karyotype group, but no significant change was found after day 8 in any karyotype subgroup or any miR.

**Table 6:** miR expression changes in peripheral blood PFP during the 1st month of chemotherapy

Subgroups	miR	miR expression changes between sampling dates log_2_FC (adjusted p)		
		Day 8–day 0	Day 15–day 0	Day 33–day 0
Whole cohort	miR-128-3p	− 2.86 (3.6*10^−7^)	− 3.15 (3.87*10^−7^)	− 3.60 (7.77*10^−10^)
	miR-181a-5p	− 1.33 (3.12*10^−2^)	− 0.93 (NS)	− 2.08 (1.01*10^−3^)
	miR-181b-5p	− 1.75 (1.48*10^−2^)	− 2.39 (2.05*10^−3^)	− 2.76 (8.78*10^−5^)
	miR-222-3p	− 1.25 (1.66*10^−2^)	− 1.03 (NS)	− 1.94 (2.09*10^−4^)
Karyotype: normal^a^	miR-128-3p	− 2.49 (2.87*10^−3^)	− 2.29 (3.09*10^−2^)	− 2.99 (4.15*10^−4^)
	miR-181a-5p	− 2.58 (2.87*10^−3^)	− 1.79 (NS)	− 3.82 (1.16*10^−5^)
	miR-181b-5p	− 2.72 (2.87*10^−3^)	− 3.44 (1.51*10^−3^)	− 4.50 (7.63*10^−7^)
	miR-222-3p	− 1.23 (NS)	− 0.98 (NS)	− 2.45 (2.41*10^−3^)
Karyotype: t(12;21)	miR-128-3p	− 4.33 (2.20*10^−8^)	− 4.62 (1.74*10^−8^)	− 4.42 (2.12*10^−8^)
	miR-181a-5p	− 1.72 (NS)	− 0.71 (NS)	− 1.13 (NS)
	miR-181b-5p	− 2.49 (4.11*10^−2^)	− 2.42 (NS)	− 2.02 (NS)
	miR-222-3p	− 2.15 (1.16*10^−2^)	− 1.59 (NS)	− 1.82 (3.39*10^−2^)
Karyotype: hyperdiploid	miR-128-3p	− 1.39 (NS)	− 2.21 (NS)	− 3.31 (NS)
	miR-181a-5p	− 1.16 (NS)	− 0.16 (NS)	− 1.01 (NS)
	miR-181b-5p	0.82 (NS)	− 0.98 (NS)	− 1.42 (NS)
	miR-222-3p	0.08 (NS)	− 0.20 (NS)	− 1.47 (NS)

*NS* no significant correlation

^a^Normal karyotype: no alteration on FISH, DNA index diploid and cytogenetics normal or unsuccessful

### Association of miR qPCR results with other MRD methods and prognostic factors

In order to further examine the value of the 4 selected miRs as MRD biomarkers, we tested their associations with results of other MRD measurement methods and with various risk factors: induction day 15 bone marrow flow cytometry MRD (the gold standard method to measure early treatment response in ALL-IC studies), day 8 absolute blast count in peripheral blood film by microscopic cytomorphology (also recorded in the ALL-IC and BFM studies), risk group stratification (based on age, initial white blood cell count (WBC), cytogenetics and early treatment response as per ALL-IC 2009) as well as end of induction absolute lymphocyte count (recently suggested as a prognostic marker) [43, 44]. All these questions were analyzed in the whole ALL group and in cytogenetic subgroups. See the significant associations highlighted in Table 7 and further results and details in Additional file 1.

**Table 7:** Associations between miR expression changes and MRD parameters or risk factors

miR	Time points	Fold change	Independent variable	Patient subgroup	Pearson’s r	Adjusted p
miR-128-3p	Day 8 vs day 0	0.107	Flow cytometry MRD in day 15 bone marrow	Whole cohort	0.88	2.71*10^−4^
miR-222-3p	Day 8 vs day 0	0.267	Flow cytometry MRD in day 15 bone marrow	Whole cohort	0.81	2.99*10^−3^
miR-128-3p	Day 8 vs day 0	0.149	Flow cytometry MRD in day 15 bone marrow	Normal karyotype^a^	0.99	7.53*10^−4^
miR-222-3p	Day 8 vs day 0	0.270	Flow cytometry MRD in day 15 bone marrow	Normal karyotype^a^	0.97	2.74*10^−2^
miR-222-3p	Day 8 vs day 0	0.190	Absolute peripheral blast count on day 8	t(12;21)	0.96	4.63*10^−2^
miR-181b-5p	Day 33 vs day 0	0.061	Absolute lymphocyte count on day 33	Normal karyotype^a^	0.95	2.98*10^−2^
miR-222-3p	Day 33 vs day 0	0.156	Absolute lymphocyte count on day 33	Normal karyotype^a^	0.94	3.87*10^−2^

In these analyses, the dependent variable is the normalized miR expression change between the time points compared, all measured in PB PFP. Fold change indicates change in the normalized miR expression between the two time points, the median of these values are provided. Pearson’s r indicates the correlation between the dependent and the independent variables. Adjusted p refers to the statistical significance of the Pearson’s correlation

^a^Normal karyotype: no alteration on FISH, DNA index diploid and cytogenetics normal or unsuccessful

All patients were MRD negative on day 33 bone marrow flow cytometry, hence this association could not be analyzed. To test if the blood WBC influences PB PFP miR expression causing bias, we checked this association on day 0 and day 33 and found no correlation.

**Fig. 6:**
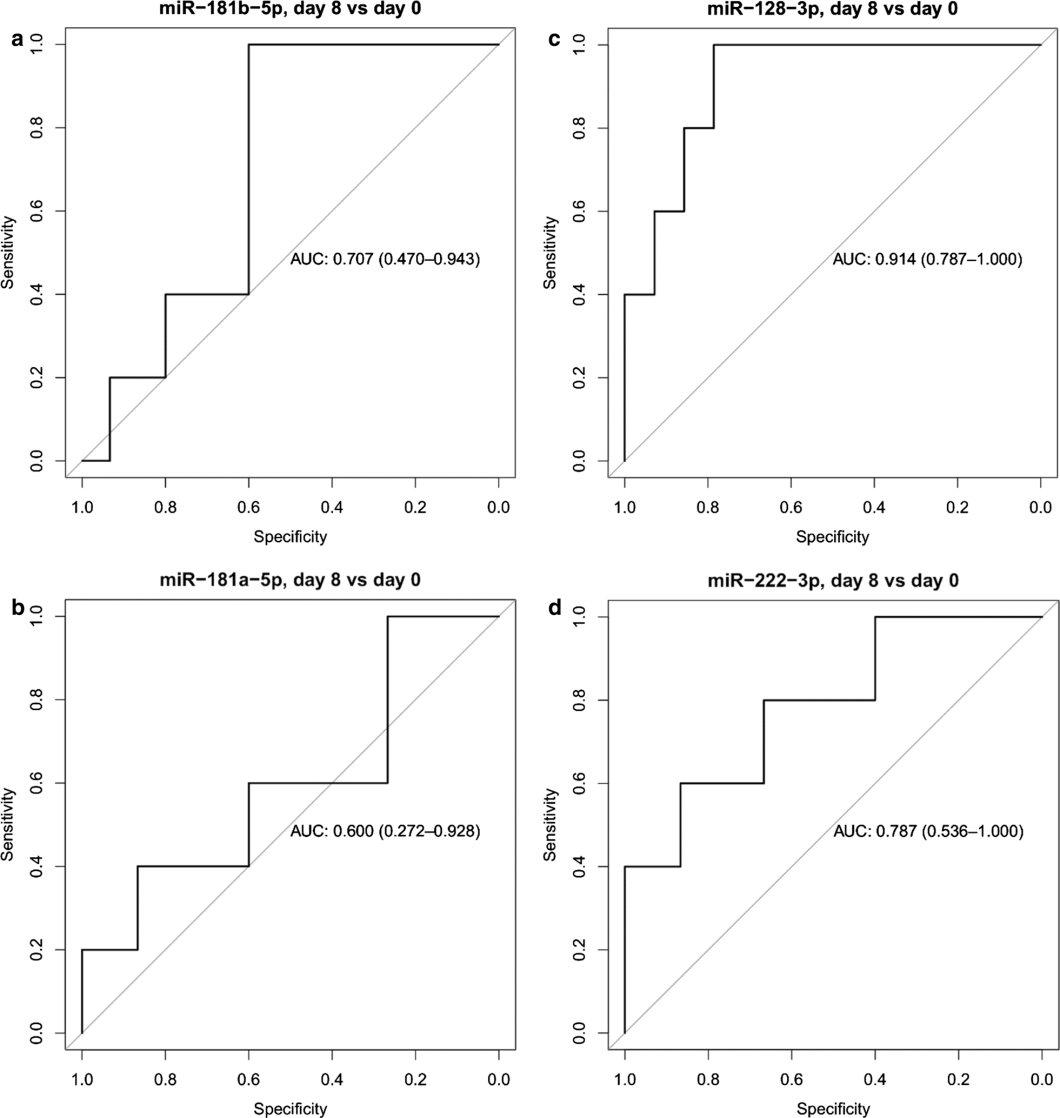
ROC curve analyses of miR expression changes between day 0 and 8. ROC curves showing the predictive accuracy of miR expression changes between day 0 and 8 to differentiate patients with high and low MRD. Area under the ROC curve (AUC) and its confidence interval are indicated. Patients with high MRD are defined as induction day 15 bone marrow flow cytometry MRD ≥ 1%. **a** miR-181b-5p; **b** miR-181a-5p; **c** miR-128-3p; **d** miR-222-3p

The predictive accuracy of microRNAs to differentiate patients with high MRD (defined as induction day 15 bone marrow flow cytometry MRD greater than 1%) versus low MRD (< 1%) was also assessed by ROC analysis and by calculating the area under the receiver operating characteristic curve (AUC). MiR-128-3p had the highest predictive accuracy (AUC = 0.91) and miR-222-3p showed also high predictive accuracy (AUC = 0.79) (Fig. 6). See the full result set of ROC analyses in Additional file 2.

## Discussion

We hypothesized that circulating miRs may be used as biomarkers of residual leukemia with the advantage of less invasive sampling for follow-up, compared to bone marrow aspirate based tests in present practice. We focused on pediatric precursor-B cell ALL patients.

Platelet-free plasma (PFP) derived from peripheral blood (PB) was chosen for analyses. In the first step, 46 candidate miRs were examined on a discovery population of de novo plus relapsed ALL patients and controls. Four miRs were selected for further analyses based on higher expression compared to controls and based on their decreasing expression by the end of induction: miR-128-3p, miR-181a-5p, miR-181b-5p and miR-222-3p. These four miRs were then tested by advanced qPCR at 4 time points along the induction cycle of an extended cohort of de novo ALL patients. Over the first week, the expression of all four miRs dropped significantly but there was no relevant further decrease at later time points. Correlation of miR expression with standard MRD results and risk factors could be demonstrated. On ROC analyses, the drop of normalized miR expression (day 8 or day 15 compared to day 0) performed better than the normalized expressions themselves at any given time points. Day 8 miR expressions performed somewhat better than day 15 miR expressions. After all, the day 0 to day 8 change in normalized expression of miR-128-3p and miR-181b-5p came out as best MRD-biomarkers.

To summarize our results, the selected circulating miRs can indeed be used as biomarkers of residual leukemia burden but are less sensitive than the day 15 bone marrow flow cytometry MRD measurement, the standard response measurement in ALL-IC. We know it from flow cytometry (as well as IgH and TCR based qPCR) MRD measurements that in reality, a monotonous multiple-log reduction occurs in the bone marrow blast percentage from day 0, through day 8 and day 15 to day 33 along the induction cycle [45–47]. By blood PFP miR measurement in our study, only a day 0 to day 8 reduction could be demonstrated. The most plausible explanation is that the overexpression of miRs originated from residual leukemic cells is measurable in the blood plasma but the noise (baseline miR expressions and their variability) is too high to allow for a sensitive leukemia follow-up. Indeed, the expression of the same miRs could be detected in the serum of healthy adults [48]. It is difficult to tell what this background noise is related to. The general understanding about miRs is that these participate in many regulation pathways in normal physiology. In microRNA Target Prediction Database (miRDB.org), an online database miR-128-3p has 1254 predicted targets while miR-222-3p has 619, miR-181a-5p and miR-181b-5p have 1408 predicted targets [49].

Our results are in parallel with findings of multiple studies which examined miR expression in leukemic cells [3, 34, 35, 50–55]. In these studies, miRs were extracted from whole bone marrow or from whole blood of pediatric- or partly pediatric-ALL patients, from mononuclear cells separated of the above samples, or from ALL cell lines. Very different sets of miRs were investigated, all partially overlapping with our selection. The 4 miRs chosen in our study were unanimously described as being overexpressed in ALL blasts. The reported rate of overexpression compared to healthy controls or to ALL patients in remission spread between 1.3 and 500 times, with very diverse normalizing RNAs used. The findings supported by most evidence are the overexpression of miR-128 and miR-181-family members. Many other overexpressed miRs were reported in ALL, e.g. let-7b, miR-222, miR-181b [31, 34].

Among the cellular miR studies, some reported different miR expression patterns in ALL cytogenetic subgroups [31, 35, 36, 50], which could not be demonstrated in our present study in PFP. Interestingly, authors disagree if the miR expression pattern of bone marrow and peripheral blood are similar [50, 51] or different [52] in intrapersonal comparisons. We did not find strong correlation between marrow PFP and blood PFP miR expressions in our TLDA results (adjusted p > 0.05, data not shown).

Three publications are available that evaluated circulating miRs in pediatric ALL. These studied plasma or serum unlike our present study (PFP samples). The identified miRs are different from ours and from each other, however, all three papers conclude that circulating miRs offer a very sensitive and specific diagnostic tool to differentiate pediatric precursor-B cell ALL cases from healthy controls [31, 56, 57]. We are not aware of any previous study which would have evaluated PFP miR expression in pediatric ALL, or would have evaluated circulating miRs as ALL MRD biomarkers. There are encouraging such publications on patient cohorts of other malignancies, though [58, 59].

Our study has special strengths when compared to other papers. A possible bias by platelets—as massive sources of circulating miRs—could be ruled out because no association of leukemia MRD and platelet originated miRs was found despite the wide range of platelet count of our patients. As no correlation could be detected between PFP miR expression levels and WBC either on day 0 (all patients with full blown ALL, before treatment) or day 33 (all the patients were MRD negative with bone marrow flow cytometry), we are convinced that the measured miR expressions were not primarily influenced by healthy leukocytes or blasts in the peripheral blood.

Mir-181 might contribute to leukemogenesis and relapse [35], it is the inducer of Wnt/β-catenin signaling pathway. The uncontrolled pathway causes leukemia by stimulating cell proliferation. It has been demonstrated using ALL-derived cell lines and bone marrow samples of patients with ALL that miR-181a-5p targets and inactivates Wnt antagonist WIF1 (Wnt inhibitory factor-1) [60].

Regarding the biology in the background, miR-128 was found to inhibit or promote tumor proliferation in several tumors and its proved target genes alter cancer-related biological processes [61]. Its expression was higher in ALL cell lines [51], in patients with poor-prognosis ALL [51], in bone marrow samples of children with ALL at diagnosis, at relapse, but not in remission [35]. Expression of miR-128 decreased during therapy [51]. One of the predicted putative targets of miR-128b is BMI1, a transcriptional factor which is important in hematopoietic stem cells and leukemia stem-cell self-renewal [35, 61]. In silico predictions suggest that miR-128 and miR-181 are among the major regulators of lymphoid differentiation. These miRs may uphold early progenitor cells at early stem-progenitor stage, prevent their further differentiation towards more mature cells by regulating molecules having crucial role in early steps of hematopoiesis. MiR-222, most likely, blocks hematopoietic differentiation at terminal stages [62]. Chromosomal location of miR-222 is close to Xp11.3 and its target is *C*-*KIT*’s 3′UTR. C-kit is a tyrosine kinase receptor with a role in cell differentiation and growth. It is expressed in hematopoietic stem cells and progenitor cells [63].

Lacking standards regarding normalizing miRs and only partial correlation of various PCR based methods which are widely known difficulties of RNA and miR lab methodologies contribute to the limitations of this paper. Still, we were able to prove our points via both methods applied. Our sample collection started in 2016, so it was not possible to correlate miR expressions to patient survival yet, and the cohort would also be small for that analysis. Given the candidate miR approach used, the existence of a better miR biomarker of leukemia MRD cannot be ruled out.

## Conclusion

In summary, circulating miR-128-3p and miR-181b-5p expression behave as biomarkers of residual leukemia in pediatric ALL. However, these are less sensitive than current standards of patient follow-up.

## Supplementary information

**Additional file 1.** Associations between miR expression changes and MRD parameters or risk factors. In these analyses, the dependent variable is the normalized miR expression change between the time points compared, all measured in PB PFP. Fold change indicates change in the normalized miR expression between the two time points, the median of these values are provided. Pearson’s r indicates the correlation between dependent and independent variables. Adjusted p refers to the statistical significance of the Pearson’s correlation. *Normal karyotype: no alteration on FISH, DNA index diploid and cytogenetics normal or unsuccessful.

**Additional file 2.** ROC curve analysis. The predictive accuracy of miRs to differentiate patients with high MRD (defined as induction day 15 bone marrow flow cytometry MRD greater than 1%) versus low MRD (< 1%) was assessed by ROC analysis and by calculating the area under the receiver operating characteristic curve (AUC).

## Data Availability

The datasets used and/or analysed during the current study are available from the corresponding author on reasonable request.

## Abbreviations

ABC: absolute blast count
ALC: absolute lymphocyte count
ALL: acute lymphoblastic leukemia
BM: bone marrow
FC: flow cytometry
FISH: fluorescent in situ hybridisation
GA: general anaesthesia
HD: hyperdiploid
IgH: immunoglobulin heavy chain
miR: microRNA
MRD: minimal residual disease
mRNA: messenger RNA
PB: peripheral blood
PCR: polymerase chain reaction
PFP: platelet-free plasma
RNA: ribonucleic acid
RNase: ribonuclease
TCR: T cell receptor
TLDA: TaqMan low density array
WBC: white blood cell
WIF1: Wnt inhibitor factor-1
